# Volumetric analysis after caries excavation with caries detecting dyes and chemomechanical caries removal agents using 3D scanner-a randomised clinical trial

**DOI:** 10.1186/s12903-024-03907-5

**Published:** 2024-02-01

**Authors:** Roja Bastia, Shashirekha Govind, Ali A. Assiry, Noura Abdulaziz Alessa, Mohammed Abdul Kader, Adbul Habeeb Adil, Mohmed Isaqali Karobari

**Affiliations:** 1 Department of Conservative Dentistry and Endodontics, Institute of Dental Science, Siksha’ O’ Anusandhan (Deemed to be) University, Bhubaneswar, Odisha 751003 India; 2 Preventive Dental Science Department, Faculty of Dentistry, Najran University, Najran, Saudi Arabia; 3 Department of Pediatric Dentistry and Orthodontics, Dental College, King Saud University, Riyadh, Saudi Arabia; 4 Department Restorative Dental science, College of Dentistry, King Khalid University, Abha, Saudi Arabia; 5 Dental Research Unit, Center for Global Health Research, Saveetha Institute of Medical and Technical Sciences, Saveetha Medical College and Hospitals, Saveetha University, Chennai, India; 6 Department of Restorative Dentistry & Endodontics, Faculty of Dentistry, University of Puthisastra, Phnom Penh, Cambodia

**Keywords:** Dental caries, Volumetric analysis, Caries detecting dye, 3D scanner, Chemomechanical caries removal

## Abstract

**Aim:**

This research aimed to use an extra-oral 3D scanner for conducting volumetric analysis after caries excavation using caries-detecting dyes and chemomechanical caries removal agents in individuals with occlusal and proximal carious lesions.

**Methods:**

Patients with occlusal (A1, A2, A3) and proximal carious lesions (B1, B2, B3) were treated with the conventional rotary technique, caries detecting dyes (CDD) and chemomechanical caries removal (CMCR) method on 90 teeth (*n* = 45 for each). Group A1, B1: Excavation was performed using diamond points. Group A2, B2: CDD (Sable Seek™ caries indicator, Ultradent) was applied and left for 10 s, and then the cavity was rinsed and dried. For caries removal, diamond points or excavators were used. Group A3 and B3: BRIX3000 papain gel was applied with a micro-brush for 20 s and was activated for 2 min, and then the carious tissue was removed with a sharp spoon excavator. Post-excavation cavity volume analysis was performed using a 3D scanner. The time required and the verbal pain score (VPS) for pain were scored during excavation. Post-restoration evaluation was performed at 1, 3, and 6 months FDI (Federation Dentaire Internationale) criteria.

**Results:**

Comparison of age, time and volume with study groups were made using Independent Sample’ t’ test and one-way analysis of variance (ANOVA) for two and more than two groups, respectively. Using Cohen’s Kappa Statistics, evaluators 1 and 2 agreed on caries removal status aesthetic, functional and biological properties at different follow-ups. The chi-square test revealed that the rotary groups [A1(2.5 ± 0.4 min) B1(4.0 ± 0.4 min)] had significantly less (*p* = 0.000) mean procedural time than CDD [A2(4.5 ± 0.4 min) B2(5.7 ± 0.4 min)] and CMCR [A3(5.4 ± 0.7 min) B3(6.2 ± 0.6 min)] groups. The CMCR group showed better patient acceptance and less pain during caries excavation than the rotary and CDD groups. CMCR group showed significantly less mean caries excavated volume(*p* = 0.000). Evaluation of restoration after 1-, 3-, and 6-month intervals was acceptable for all the groups.

**Conclusion:**

Brix3000 helps effectively remove denatured teeth with less pain or sensitivity. The time required for caries removal was lowest in the rotary method and highest in the brix3000 group, while the volume of caries removed was the lowest for brix3000 and highest for the rotary group.

## Introduction

“Caries” is a combination of the Latin word for “rot” and the Greek word for “death,” “ker.” The World Health Organization (WHO) defines " dental caries” as a “localized pathological process of external origin, involving softening of hard tooth tissue and progressing to the formation of a cavity“ [1]. Carious dentine is divided into two distinct layers, each with unique ultramicroscopic and chemical characteristics. Carious dentine’s outer layer (infected dentine) is irreversibly denatured, infected with bacteria, and ungeneralizable and must be removed. Carious dentine’s inner layer (affected dentine) is reversibly denatured, infected, and mineralizable and should be preserved [2]. Visual inspection is one of the dentists’ most prevalent diagnostic procedures [3]. The need for early identification of caries was emphasized during the International Consensus Workshop on Caries Clinical Trials, and the notion of developing an International Caries Identification and Assessment System (ICDAS) was advocated. According to research, ICDAS provides trustworthy and accurate findings in identifying early caries lesions and long-term alterations [4, 5].

Traditional detection methods rely on visual and tactile sensations; however, these procedures are subjective and vary across practitioners [6]. When removing carious tissue with handpieces and burs, there is a tendency to over-excavate [7]. Different colours differentiate between healthy and decaying hard tissues [8]. Caries-detecting dyes are colour markers that bind to the substance they are applied to [9]. When a dye is put on the surface of a tissue or substance, it becomes embedded in the fibres, pores, or other structures [10].

Various caries detector dyes with different chemical formulas are commercially available today to help the dentist differentiate the softened dentin (Caries detector, Kuraray America Inc., Caries Finder Detection Dye, etc.) [11]. Sable Seek caries indicator (Ultradent, USA) comprises FD&C dyes in a glycol-based solution that stains weakened/infiltrated dentin green, differentiating it from pulp and enabling its discovery even in deep cavities. It stains demineralized dentin and is beneficial for difficult-to-see places such as preparation undercuts and areas near the DE (Dentino-enamel) junction) [12].

Minimally invasive dentistry (MID) is a concept that treats dental caries by changing patients’ behaviours as well as correcting cavities [13]. When a restoration is required, this viewpoint prioritizes the preservation of dental tissue [14]. Carious dentin must be removed selectively, considering the preservation of good tissue and the reduction of unpleasant stimuli [15, 16]. The chemomechanical caries treatment approach eliminates carious dentine selectively while avoiding the unpleasant and unneeded loss of sound dentine.

In 2016, a papain-based agent (Brix 3000) was launched. It is a non-toxic papain-based gel formulation derived from the latex and fruits of green papaya (Carica Papaya). The high concentration of papain (3,000 U/mg in a 10% concentration) and bioencapsulation EBE (Encapsulated Buffer Emulsion) technology provide the gel with the optimal pH to immobilize the enzyme at the moment of exerting proteolysis in collagen, thus increasing its activity [17]. Clinical efficiency or the time factor may be critical for some patients, particularly youngsters and medically impaired individuals, to accept therapy. The amount of tissue eliminated demonstrates the chemomechanical method’s low intervention impact in eliminating carious tissue.

Johnson and Craig demonstrated that adding silicone results in the best undercut recovery, exceptionally high accuracy, excellent rip resistance, reduced polymerization shrinkage, and increased dimensional stability, allowing for repeated precise castings. The polyvinyl siloxane substance has an overall accuracy of 96.86% [18].

Extra-oral 3D scanners dominate and are more accurate in volumetric tissue analysis than 2D assessment methods such as cavity wall thickness measurement [19]. In 12 s, the Medit T500 scanner can scan the whole arch. It offers automated imprint scanning on three axes. Exocad Dental DB 2.3 Matera 6990 software is then used to assess the data. This enables the processing of triangulated point cloud data representing a geometrical model. It also allows for further computations on the provided geometry.

The literature documents various studies comparing several chemomechanical agents, but there is a lack of comparison between caries excavation using dyes and chemomechanical agents. There is no study in which a 3D scanner was used to calculate volume after caries excavation. Theoretically, much attention has been placed on improving tooth structure conservation, but it is rarely applied in practice. As a result, to bridge this gap, this study was carried out to assess the efficacy of various conservative approaches in caries excavation to determine the optimum choice to be utilized frequently in clinical practice.

The aim of the study was volumetric analysis after caries excavation using caries-detecting dyes and chemomechanical caries removal agents in patients having occlusal and proximal carious lesions using an extra-oral 3D scanner. The time required, and pain perception during caries excavation were recorded. Post-restoration assessment at 1, 3 and 6 months using (Federation Dentaire Internationale) FDI criteria was also assessed. The null hypothesis states no difference between caries-detecting dyes and chemomechanical caries removal agents in volume measured after caries excavation.

## Materials and methods

The current study was carried out at the Department of Conservative Dentistry and Endodontics, Institute of Dental Sciences, Bhubaneshwar, Odisha, India, in accordance with the principles of the Declaration of Helsinki, 2008. Before starting the study, approval of the research protocol and ethical clearance was obtained from the institutional review board, Siksha’ O’ Anusandhan (deemed to be) University Ref.no/IEC/IMS.SH/SOA/2022/451. The study was registered in clinical.trials.gov on 31/01/2022 (CTRI/2022/01/039870) and followed the Consolidated Standards of Reporting Trials 2010 guidelines. A detailed medical and dental history was recorded.

Inclusion criteria: Class I and Class II carious lesions in permanent premolars and molars with maximum entrance size of ≥ 3 mm, soft consistency, and a brownish-grey discolouration; Caries categorized as D2 under ICDAS classification; Age 17–45 years including both genders (Male and Female); Co-operative patients approving the trial.

Exclusion criteria: Pulpal/Periapical pathology; Patients with underlying medical complications; Periodontal problems; non-vital teeth; Fractured teeth.

A single operator screened patients by clinical examination with the help of a mouth mirror and a World Health Organization (WHO) periodontal probe followed by a radiographic examination. After radiographic examination, patients were scored according to ICDAS scoring criteria. According to ICDAS scoring, carious lesions extending to D2 were included in the study.

A duration of two weeks was dedicated to conducting comprehensive training, during which the evaluators, as well as operators, engaged in the clinical examinations. Through this training, they acquired expertise in investigating the cavity by eliminating incomplete and total caries. Post-screening procedure, patients were subsequently grouped randomly using computer-generated software (accessed at www.randomizer.org.). The numbers were distributed by a researcher who was not involved in the clinical phase.

Using G.Power-3.1.9.4 (accessed on 5-11-2021), sampling was computed. Effect size w:0.4, α err prob – 0.05, power (1-β err prob) − 0.9 [2]. The total sample size was 97; following the requirements of the study, a total of 90 samples were considered, of which 45 were from each group.

**Fig. 1:**
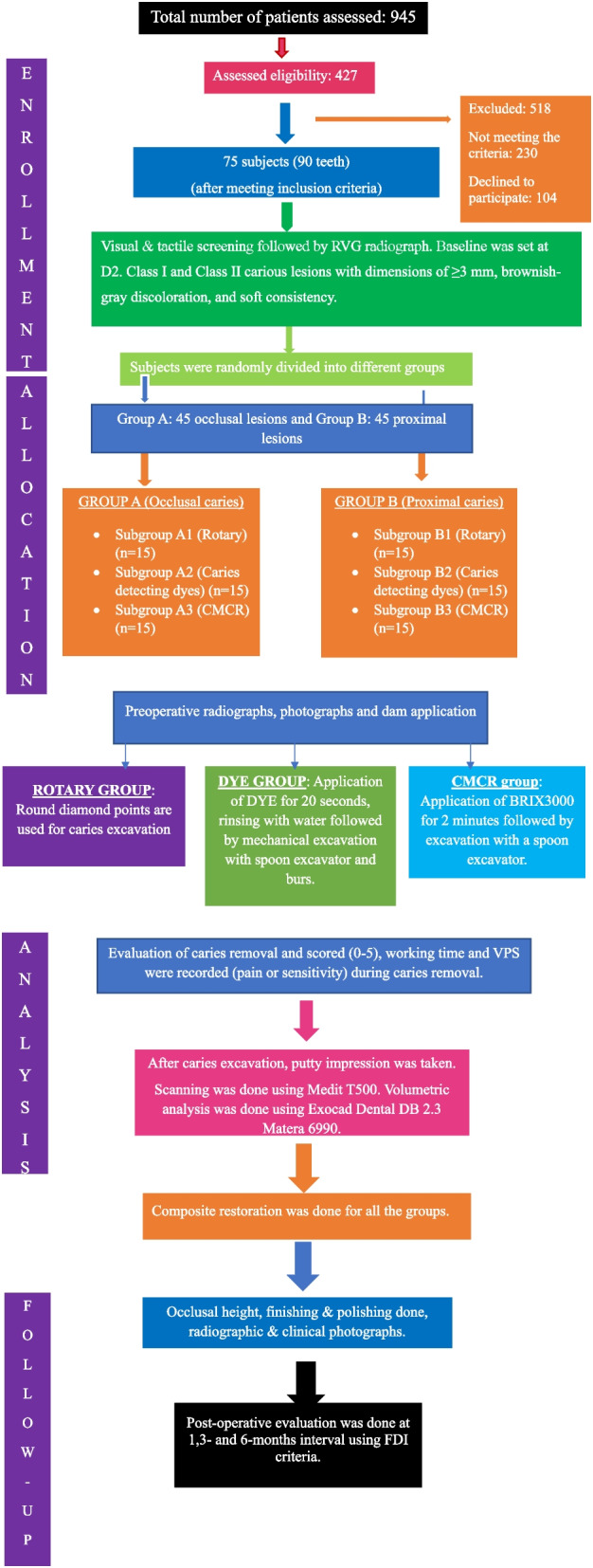
Flow chart of clinical study (CONSORT)

The randomization process yielded a total of 90 teeth obtained from patients exhibiting either a proximal (P) or an occlusal (O) carious lesion. Moreover, Group A had 45 occlusal lesions, while Group B had 45 proximal lesions. (Fig. 1)

### Treatment protocol

Clinical photographs were taken before, during, and after the restoration procedure. Bitewing (Radiovisiography)RVGs were captured using NanoPix1, Intraoral Digital Imaging Sensor, which utilizes (Active Pixel Sensor) APS CMOS technology and is manufactured by Eighteeth, Caretechion GmbH, Duesseldorf. Additionally, silicon impressions of the pre-excavated lesions were made using Extreme Putty, manufactured by Medicept UK LTD, Middlesex, UK. These impressions were used to calibrate the extent of caries.

A single trained operator commenced the surgery, and local anaesthetic (Lidayn, Lidocaine Topical Aerosol USP 15% w/w) was administered as required.

Two measurements were made with the Api (Universal) UNC15 periodontal probe. The bucco-lingual and mesio-distal measurements of the carious lesion were assessed, while the depth of the lesion was determined at three distinct regions, preferably before excavation, to determine the extent of pulpal involvement.

Following the cavity evaluation using a periodontal probe, silicon impressions (Extreme Putty) were taken to measure the cavity depth after excavation. These impressions were utilized to calculate the volume of the cavity.

### Caries excavation and treatment time

The carious lesions in both the occlusal (Group A) as well as proximal (Group B) groups were treated in the control group (Group A1, B1) utilizing 440 S, 462R, and 440 diamond points (Shofu Inc Diamond Points Fg, Kyoto, Japan) as well as an air-rotor handpiece (NSK PANA-AIR MB2 CB90036, Japan) operating at range of 100,000–150,000 rpm.

In Groups A2 and B2, caries-detecting dye (Sable Seek™ caries indicator, Ultradent) was applied with the help of a micro-brush on the carious lesions. It was left for about 10 s, and subsequently, the cavity was rinsed and air-dried. A slow-speed round bur 440 S, 462R, and 440 diamond points (Shofu Inc Diamond Points Fg) or excavator (Mcare Instrument, XmsH, Maharashtra, India) was used for caries removal. It was repeated until caries were removed entirely.

For Groups A3 and B3, BRIX3000 papain gel (Brix Medical Science, Carcañá, Argentina) solution was applied using a micro-brush (20 s) and was activated for 2 min, and then the carious tissue was extracted using a sharp spoon excavator (Mcare Instrument). The solution was repeatedly applied multiple times till successful eradication of caries was achieved.

Following the excavation of cavities, two investigators assessed the caries removal by adopting the Ercison D et al. (1999) scale for caries assessment, which assigns scores ranging from 0 to 5 [29]. The working duration was recorded utilizing a digital stopwatch. The digital stopwatch was initiated at the commencement of excavation or cavity preparation while stopped upon it.

### Measurement of the volume of carious tissue excavated

**Fig. 2:**
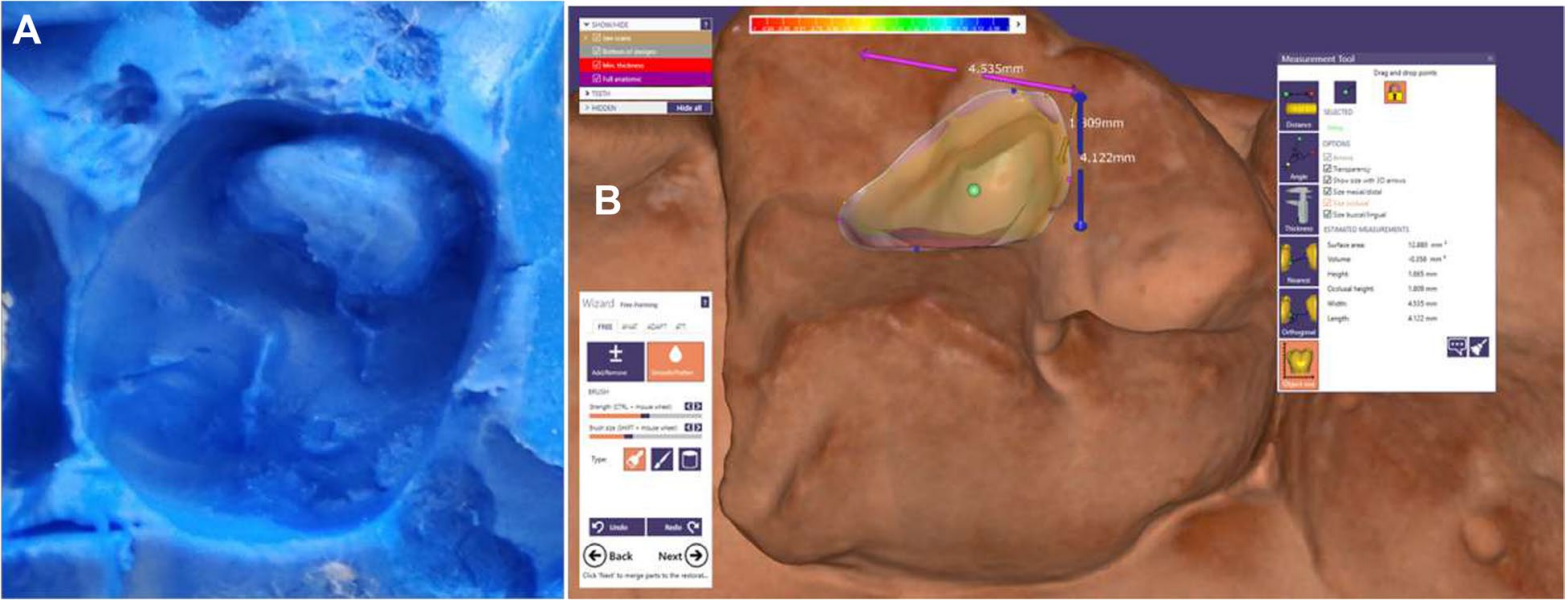
The steps of volume determination by 3D scanner. **A** Silicone impression (**B**) Volume determination illustration using exocad software

Depending on the intended application, putty single-step elastomeric addition silicone material (Extreme Putty, Medicept UK LTD, Middlesex, UK) produced post-caries excavation impressions in a sectioned impression tray. The initial and final cavity volume disparities were assessed using a 3D scanner, namely the MEDIT T500 (MEDIT Corporation, 23 Goryeodae-ro 22 Gil, Seongbuk-gu, Seoul, South Korea). Exocad Software, specifically the exocad Dental DB 2.3 Matera 6990 version developed in Darmstadt, Germany, was also employed for data analysis (see Fig. 2). The 3D structure of the excavated cavity was acquired through a 3D laser scanning microscope employing a phase-shifting optical triangulation technique, along with an LED light source (150 ANSI-Lumens, blue LED).

In the prepared surfaces, the accompanying software does an automated computation of the enclosed volume. This calculation was done by choosing a point spacing of 0.04 mm and scanning an area measuring 100 mm × 73 mm × 60 mm.

### Pain assessment

The same investigator documented the pain assessment during therapy for each patient according to the Verbal Pain Score (Kocher et al. 2011). Local anaesthesia (Lidayn, Lidocaine Topical Aerosol USP 15% w/w) was administered based on the patient’s response. 0-No pain, 1-Mild pain (Pain recognizable but no discomfort), 2- Moderate pain (Pain discomforting but bearable), 3-Severe pain (Pain that causes considerable discomfort and is difficult to bear), 4- Very severe pain.

### Restoration

The cavity was completed in all groups using a micro-grained aluminium oxide abrasive Arkansas stone (RD2 &TC1; Shofu Dura-White Stones, Kyoto, Japan) at 5000 to 20,000 rpm while maintaining consistent contact pressure and disinfected with gluma desensitizer (Heraeus Kulzer Germany).

The occlusal cavities were treated by employing a bonding agent (Tetric®N-Bond Universal, Ivoclar Vivadent, Schaan, Liechtein) as well as a bulk fill composite material (Tetric® N-Ceram Bulk Fill, Ivoclar Vivadent, Schaan, Liechtein). The composite material was curated using a light-curing device (Bluephase®N Ivoclar Vivadent, Schaan, Liechtein) in the soft mode for 20 s. Additionally, the sandwich technique was utilized in cases where the cavities were deep and close to the pulp, using Ionosit (DMG CPF GmbH, Hamburg, Germany).

The proximal caries group had the proximal wall replaced using a sectional matrix band (TDV Special Matrices Kit, TDV Dental Ltd. Pomerode/SC, Brasil). The restoration of cavities involved using a bonding agent (Tetric®N-Bond Universal) and bulk fill composite material (Tetric® N-Ceram Bulk Fill). Subsequently, the composite was cured for 20 s. In cases where the cavities were deep and close to the pulp, a sandwich technique was employed using Ionosit (DMG CPF GmbH).

After that, occlusion correction and finishing and polishing restorations were carried out for both groups using the Shofu Composite Finishing and Polishing Kit.

### Follow up

The post-restorations were examined by two expert calibrated evaluators utilizing mirrors, probes, clinical pictures, and radiographs at one month, 3 months, and 6 months, per the FDI evaluation criteria. The scale used in this study encompasses 16 distinct categories, each rated on a scale ranging from 1 to 5. These categories encompass various aspects, such as aesthetic, functional, and biological qualities. The scoring factors utilized in this study were as follows: 1—clinically excellent/very good, 2—clinically good, 3—clinically sufficient/satisfactory, 4—clinically unsatisfactory, and 5—clinically inadequate.

###  Statistical analysis

Data collected during the study were scrutinized, codified, and entered into the IBM SPSS Statistics, 24.0 software, www.spss.co.in, for analysis. The categorical variables like age group, Gender, M/D, pain score, caries removal status, aesthetic properties, and functional and biological properties at different follow-ups were done using a frequency distribution procedure. Their association with study groups was studied using the Chi-square test of independence. Comparisons of age, time, and volume with study groups were made using an independent sample t-test and one-way analysis of variance (ANOVA) for two and more than two groups, respectively. Using Cohen’s Kappa Statistics, the agreement of evaluators 1 and 2 of caries removal status (was 0.62 to 0.68), aesthetic, functional and biological properties at different follow-ups. Cohen’s kappa is a measure that indicates to what extent two ratings agree better than chance level. The cut-off value ‘p’ <0.05 was considered to indicate statistical significance.

## Results

### Demographic profile

Table 1 presents the distribution of cases by age and gender by two groups, i.e., occlusal and proximal caries. Of the 90 patients, 43.3% belonged to < 30 years of age, 37.8% to 30–40 years and 18.9% to > = 40 years. The majority of the patients are in the middle and younger age groups. The age was not significantly associated with occlusal and proximal group (*p* = 0.348). Males are more prevalent among the patients, with a share of 63.3%. The gender distribution did not significantly affect the caries group (*p* = 0.827).

**Table 1:** Demographic profile by groups

Age group	Caries Group				Total		χ2, *p*
	Occlusal caries		Proximal caries				
	No.	%	No.	%	No.	%	
< 30	22	48.9	17	37.8	39	43.3	χ2=2.112 *p* = 0.348
30–40	17	37.8	17	37.8	34	37.8	
≥ 40	6	13.3	11	24.4	17	18.9	
Mean ± SD	30.2 ± 7.5		32.9 ± 7.4		31.5 ± 7.6		0.081^a^
Gender							
Male	29	64.4	28	62.2	57	63.3	χ2=0.048 *p* = 0.827
Female	16	35.6	17	37.8	33	36.7	
Total	45	100	45	100	90	100	

^a^Independent Sample ‘t-test ‘*p*-value

### Volume and time

#### In the occlusal group

The mean volume was highest in the rotary method and lowest in the BRIX 3000 group. The difference among the mean volumes was significant (*p* = 0.000) within the occlusal group. The mean time of caries removal was lowest in the rotary method and highest in caries-detecting dyes (Table 2).

**Table 2:** Comparison of time and volume within Occlusal caries group

Variables	Occlusal caries group						ANOVA ‘*p*’ value
	Rotary (*N* = 15)		Caries detecting dyes (*N* = 15)		BRIX3000 (*N* = 15)		
	Mean	SD	Mean	SD	Mean	SD	
Time (in min)	2.5	0.4	5.4	0.7	4.5	0.4	0.000
Volume (mm^3^)	3.2	0.3	1.8	0.4	1.4	0.4	0.000

#### In the proximal group

The mean volume was highest in the rotary method and lowest in the BRIX 3000 group. The mean volume was 16.4 ± 5.5mm^3^ in the detecting dye group. The difference among the mean volumes was significant (*p* = 0.000) within the proximal group. The mean time of caries removal was lowest in the rotary method and highest in caries-detecting dyes (Table 3).

**Table 3:** Comparison of time and volume within the proximal caries group

Variables	Proximal caries group						ANOVA ‘*p*-value
	Rotary (*N* = 15)		Caries detecting dyes (*N* = 15)		BRIX3000 (*N* = 15)		
	Mean	SD	Mean	SD	Mean	SD	
Time (in min)	4.0	0.4	5.7	0.4	6.2	0.6	0.000
Volume (mm^3^)	25.8	5.9	16.4	5.5	15.2	3.3	0.000

### Association of pain score with caries removal method within the occlusal and proximal groups

**Fig. 3:**
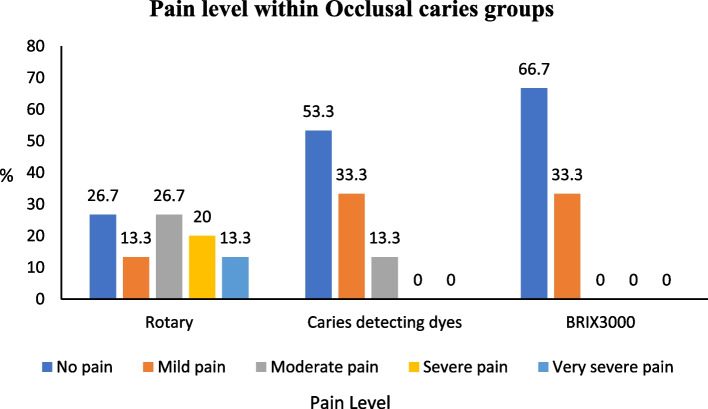
Association of pain score within occlusal caries groups

**Fig. 4:**
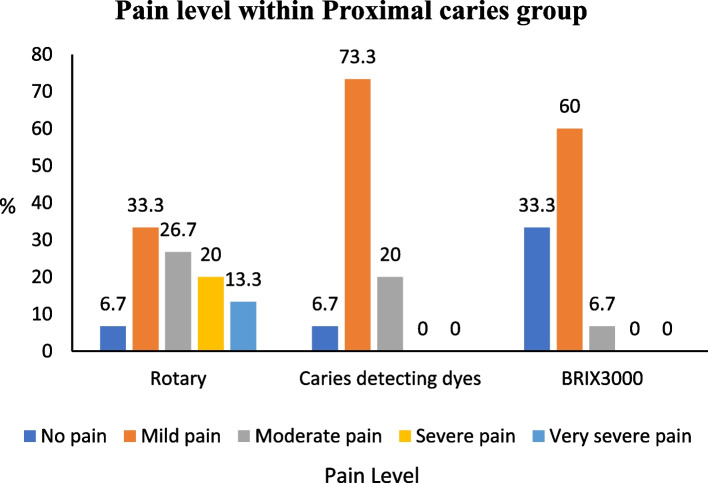
Association of pain score within proximal caries groups

In the occlusal group, the BRIX3000 method exhibited a significantly higher percentage of painless cases (66.7%) than the rotary method (26.7%). The BRIX 3000 method exhibits a notably reduced level of pain compared to caries-detecting dyes (*p* = 0.021). In the proximal group, the caries-detecting dyes group experienced mild pain in 73.3% of cases, while the BRIX3000 group experienced it in 60% of cases during the excavation process. The rotary group experienced pain ranging from moderate (26.7%) to very severe (13.3%) in the occlusal and proximal groups (Figs. 3 and 4).

### Association of aesthetic properties in occlusal group and proximal group

Regarding surface lustre, a lustre similar to enamel was achieved in 85.7% of cases at one month and in 83.3% of cases at 3 and 6 months. There were no significant differences among the methods used (*p* = 0.558 at one month, 0.301 at three months, 0.301 at six months). At one month, three months, and six months, the BRIX3000 and rotary methods showed satisfactory colour stability and translucency, with no surface staining, compared to the caries-detecting dyes method. The anatomic form was optimal for all cases at 1, 3 and 6 months for all methods (Table 4).

**Table 4:** Association of Aesthetic properties at different months within groups

	Group						Total		χ2, *p*
	Rotary		Caries detecting dyes		BRIX3000				
**Surface Luster T 1 MONTH**	**No.**	**%**	**No.**	**%**	**No.**	**%**	**No.**	**%**	
Luster comparable to enamel	12	85.7	11	78.6	13	92.9	36	85.7	χ2=1.167 p=0.558
Slightly dull, not noticeable from a speaking distance	2	14.3	3	21.4	1	7.1	6	14.3	
No surface staining	13	92.9	12	85.7	14	100	39	92.9	χ2=2.154 p=0.341
Minor staining, easily removable	1	7.1	2	14.3	0	0	3	7.1	
Good color match. No difference in shade and translucency	14	100	12	85.7	14	100	40	95.2	χ2=4.200 p=0.122
Minor deviations	0	0	2	14.3	0	0	2	4.8	
Form is ideal	14	100	14	100	14	100	42	100	-
**AT 3 MONTHS**									
Luster comparable to enamel	12	85.7	10	71.4	13	92.9	35	83.3	χ2=2.400 p=0.301
Slightly dull, not noticeable from speaking distance	2	14.3	4	28.6	1	7.1	7	16.7	
No surface staining	13	92.9	12	85.7	14	100	39	92.9	χ2=2.154 p=0.341
Minor staining, easily removable	1	7.1	2	14.3	0	0	3	7.1	
Good color match. No difference in shade and translucency	14	100	11	78.6	14	100	39	92.9	χ2=6.462 p=0.040
Minor deviations	0	0	3	21.4	0	0	3	7.1	
Form is ideal	14	100	14	100	14	100	42	100	-
**AT 6 MONTHS**									
Luster comparable to enamel	12	85.7	10	71.4	13	92.9	35	83.3	χ2=2.400 p=0.301
Slightly dull, not noticeable from a speaking distance	2	14.3	4	28.6	1	7.1	7	16.7	
No surface staining	13	92.9	12	85.7	14	100	39	92.9	χ2=2.154 p=0.341
Minor staining, easily removable	1	7.1	2	14.3	0	0	3	7.1	
Good color match. No difference in shade and translucency	14	100	11	78.6	14	100	39	92.9	χ2=6.462 p=0.040
Minor deviations	0	0	3	21.4	0	0	3	7.1	
Form is ideal	14	100	14	100	14	100	42	100	-
**Total**	14	100	14	100	14	100	42	100	

### Association of functional properties in occlusal caries and proximal group

In all patients with occlusal and proximal caries under all three methods at 1, 3, and 6 months, the restoration was retained with no fractures/cracks and a harmonious outline, no gaps, and no discolouration. Instead of restoration wear, the overall rate of enamel wear was noted after three and six months (Table 5).

**Table 5:** Association of functional properties at different months within the groups

	Group						Total		χ2, *p*
	Rotary		Caries detecting dyes		BRIX3000				
**AT 1 MONTH**	**No.**	**%**	**No.**	**%**	**No.**	**%**	**No.**	**%**	
Restoration retained, no fractures/cracks	14	100	14	100	14	100	42	100	-
Harmonious outline, no gaps, no discolouration	14	100	14	100	14	100	42	100	-
Physiological wear equivalent to enamel (80-120% of corresponding enamel)	9	64.3	7	50	11	78.6	27	64.3	χ2=2.489 p=0.288
Normal wear with only a slight difference to enamel (50-80% or 120-150% of corresponding enamel)	5	35.7	7	50	3	21.4	15	35.7	
Normal contact point (floss or 25 metal blades can be inserted but not 50 blades)	14	100	14	100	12	85.7	40	95.2	χ2=4.200 p=0.122
It is slightly too strong but no disadvantage	0	0	0	0	2	14.3	2	4.8	
Radiographic examination. No pathology, the harmonious transition between restoration and tooth	14	100	14	100	14	100	42	100	-
**Patient view**									
Entirely satisfied	4	28.6	5	35.7	5	35.7	14	33.3	χ2=0.214 p=0.898
Satisfied	10	71.4	9	64.3	9	64.3	28	66.7	
**AT 3 MONTHS**									
Restoration retained, no fractures/cracks	14	100	14	100	14	100	42	100	-
Harmonious outline, no gaps, no discolouration	14	100	14	100	14	100	42	100	-
Physiological wear equivalent to enamel (80-120% of corresponding enamel)	0	0	0	0	4	28.6	4	9.5	χ2=8.842 p=0.012
Normal wear with only a slight difference to enamel (50-80% or 120-150% of corresponding enamel)	14	100	14	100	10	71.4	38	90.5	
Normal contact point (floss or 25 metal blades can be inserted but not 50 blades)	14	100	14	100	12	85.7	40	95.2	χ2=4.200 p=0.122
It is slightly too strong but no disadvantage	0	0	0	0	2	14.3	2	4.8	
No pathology, the harmonious transition between restoration and tooth	14	100	14	100	14	100	42	100	-
**Patient view**									
Satisfied	14	100	14	100	14	100	42	100	-
**AT 6 MONTHS**									
Restoration retained, no fractures/cracks	14	100	14	100	14	100	42	100	-
Harmonious outline, no gaps, no discolouration	14	100	14	100	14	100	42	100	-
Normal wear with only a slight difference to enamel (50-80% or 120-150% of corresponding enamel)	11	78.6	11	78.6	12	85.7	34	81	χ2=0.309 p=0.857
Differing wear rate to enamel but within the biological variation	3	21.4	3	21.4	2	14.3	8	19	
Normal contact point (floss or 25 metal blades can be inserted but not 50 blades)	14	100	14	100	12	85.7	40	95.2	χ2=4.200 p=0.122
It is slightly too strong but no disadvantage	0	0	0	0	2	14.3	2	4.8	
No pathology, the harmonious transition between restoration and tooth	14	100	14	100	14	100	42	100	-
**Patient view**									
Satisfied	14	100	14	100	14	100	42	100	-
**Total**	14	100	14	100	14	100	42	100	

### Association of biological properties in occlusal caries and proximal group

Regardless of technique, all cases at 1, 3, and 6 months showed no signs of general or oral discomfort, no signs of inflammation, no pockets, no hypersensitivity, secondary or primary caries, complete integrity, or vitality (Table 6).

**Table 6:** Association of biological properties at different months within groups

	Group							Total			χ2, *p*
	Rotary			Caries detecting dyes		BRIX3000					
	No.		%	No.	%	No.	%	No.		%	
**AT 1 MONTH**											
No hypersensitivity, normal vitality	12		85.7	12	85.7	14	100	38		90.5	χ2=2.211 *p* = 0.331
Low hypersensitivity for a limited period of time, normal vitality	2		14.3	2	14.3	0	0	4		9.5	
Recurrence of caries. No secondary or primary caries	14		100	14	100	14	100	42		100	-
Complete integrity	14		100	14	100	14	100	42		100	-
Periodontal response. No plaque, no inflammation, no pockets	14		100	14	100	14	100	42		100	-
Adjacent mucosa. Healthy mucosa adjacent to restoration	14		100	14	100	14	100	42		100	-
Oral and general health. No oral or general symptoms	14		100	14	100	14	100	42		100	-
**AT 3 MONTHS**											
No hypersensitivity, normal vitality	12	85.7		13	92.9	14	100		39	92.9	χ2=2.154 *p* = 0.341
Low hypersensitivity for a limited period of time, normal vitality	2	14.3		1	7.1	0	0		3	7.1	
No secondary or primary caries	14		100	14	100	14	100	42		100	-
Tooth integrity. Complete integrity	14		100	14	100	14	100	42		100	-
Periodontal response. No plaque, no inflammation, no pockets	14		100	14	100	14	100	42		100	-
Adjacent mucosa. Healthy mucosa adjacent to restoration	14		100	14	100	14	100	42		100	-
No oral or general symptoms	14		100	14	100	14	100	42		100	-
**AT 6 MONTHS**											
Postoperative hypersensitivity and tooth vitality. No hypersensitivity, normal vitality	12		85.7	13	92.9	13	92.9	38		90.5	χ2=0.553 *p* = 0.759
Low hypersensitivity for a limited period of time, normal vitality	2		14.3	1	7.1	1	7.1	4		9.5	
No secondary or primary caries	14		100	14	100	14	100	42		100	-
Tooth integrity. Complete integrity	14		100	14	100	14	100	42		100	-
Periodontal response. No plaque, no inflammation, no pockets	14		100	14	100	14	100	42		100	-
Adjacent mucosa. Healthy mucosa adjacent to restoration	14		100	14	100	14	100	42		100	-
No oral or general symptoms	14		100	14	100	14	100	42		100	-
**Total**	14		100	14	100	14	100	42		100	

The dropout rate during follow-up evaluation was as follows: at 1, 3 and 6 months, patients were examined and scored as per FDI criteria. Two patients could not turn up for three months due to travel inconveniences. Four patients could not turn up for follow-up at six months due to unavoidable circumstances. It was noted that no significant difference was found due to loss of follow-up.

## Discussion

The current study used a parallel group method, occlusal and proximal caries, to examine and comprehend the procedure steps and pain perception to evaluate the traditional approach with CDD and CMCR substances. Parallel trials are versatile and straightforward, and trials have higher external validity. Studies need not focus on challenges related to carryover effect, analysis errors, period effects or washout period units amongst interventions [20]. The operator had no access to the patient’s data, while the evaluator was entitled to every single one of the patients’ responses and had been informed about the treatment that had been adopted. Occlusal caries are far more straightforward to treat compared to proximal caries, which require the elimination of impacted food, reducing gingival bleeds, easy access, growth of contact or contour, surface overlapping on X-ray, cutting of the nearby teeth (control group), preventing a gingival marginal discrepancy, as well as handling of distal proximal surface caries, that is typically difficult. This study did not significantly associate age with the occlusal and proximal caries group. Males are more prevalent among the patients. The gender distribution did not significantly affect the caries group. In literature, females display a greater caries rate than males, and one team demonstrated that adult men have a higher caries rate than females, suggesting a gender bias in obtaining or using dental care [21, 22]. These findings followed a previous study by Shaffer et al., 2015 [23].

Compared to stainless steel burs, carbide burs, and diamond points had a higher cutting efficacy and less heat production [24]. Clinicians favour the traditional rotary technique (diamond points or carbide burs) for excavating caries because it requires a shorter time. However, it has the disadvantage of over-preparing and eliminating odontoblastic reaction zone plugs, reversibly impacted dentin, and sound tooth structure, permanently exposing permeable strong dentin [25].

Rotating instruments are the most common way to remove caries. However, cavity preparation is painful and uncomfortable because of the sensitive vital pulp, pressure inside the tooth (mechanical stimulus), noise and vibrations that travel to the bone, and, lastly, elevated temperatures of the surface cut because of heat stimulation [24, 26].

None of the patients in the BRIX3000 group reported pain or received any form of anaesthetic. Nevertheless, patients from both the rotary and CDD groups, who had five occlusal caries teeth and eight proximal caries teeth, expressed a preference for LA during the excavation process. The discomfort level observed in this study is notably reduced in the BRIX3000 procedure, with caries-detecting dyes showing a similar trend in both occlusal and proximal groups. The efficacy of the BRIX3000 gel is limited to denuded fibres within demineralized dentin, preventing painful removal and injury to intact dentin [27].

Braum et al. have reported the observation of a mild anaesthetic effect resulting from applying the gel. The findings yielded comparable outcomes to those reported by Gurbuz, whose study determined that the chemomechanical technique exhibited efficacy in caries removal, resulting in less pain and diminished reliance on local anaesthesia. Consequently, this approach alleviated children’s anxiety, fear, and stress levels [28].

The process of caries excavation was shown to be more straightforward in cases where the dentinal caries were exposed, as opposed to cases where the caries were layered or covered with enamel. When dealing with proximal lesions, it was found that it was easier to address cases where there were no marginal ridges or open cavities compared to situations with an intact undermined enamel marginal ridge. The optimal caries excavation strategy involves the removal of permanently denatured tissue while preserving any possible remineralizable tissue in the cavity floor. The total assessment of caries removal was based on several criteria, including the ability of explorers to go smoothly into the dentin without encountering any resistance, the presence of a brown-hard surface, and the absence of any stains on the surface [29]. No statistically significant correlation was observed between caries removal procedures within the occlusal and proximal caries groups. The minimum size of the carious lesion chosen for this investigation was set at ≥ 3 mm. This criterion was established because lesions measuring ≤ 2 mm would not have allowed adequate instrument access to the dentinal caries [30].

The researchers Abuasi and Wassell concluded that the optimal method for capturing a putty-wash impression is using the one-stage approach, in addition to silicone putty. The current investigation utilized the single-step putty/wash impression procedure, including silicone impression material, to capture the cavity dimensions [31, 32]. The current study employed post-excavation impression methodology to conduct volumetric analysis using a 3D laboratory scanner (MeditT500). The Control groups A1 and B1 exhibited greater volume than the others, indicating that a more significant amount of sound tooth structure was lost in the Control group. In contrast, using CMCR agents allowed for the preservation of the excavation surface and the potential for remineralization.

The utilization of direct 3D scanning for impression analysis offers advantages in terms of time efficiency and accuracy in volume determination compared to scanning the cast. This is due to the additional time required for cast fabrication, the potential for contraction or expansion of the dental stone material and the inclusion of porosity, which can introduce inaccuracies in the measurements [33]. The utilization of micro-CT imaging techniques demonstrated that the conventional rotary method resulted in more post-caries excavation cavities than the CMCR technique, as reported in previous publications [34, 35]. The study found that the rotary approach had the most enormous mean volume, while the CMCR group had the lowest mean volume. In the CDD group, the mean volume was determined to be 1.8 ± 0.4. A significant difference was observed in the mean volumes within the occlusal and proximal groups (*p* = 0.000).

The rotary instrument exhibited the most incredible volume of caries removal, likely due to its tendency to prepare the cavities excessively due to diminished sensitivity to tactile feedback. Consequently, there was a substantial and fast elimination of tissue, accompanied by diminished regulation of the overall procedure. Hence, the operator did not always possess immediate clarity regarding attaining the authentic clinical endpoint. The excavation process proceeded in a more robust dentin, resulting in subsequent over-preparation [36]. Using carbide or diamond burs with high-speed instruments for cavity preparation frequently removes reversibly damaged dentin and the plugs inside the odontoblastic reaction zone, thereby exposing the comparatively more permeable healthy dentin [37].

The BRIX 3000 gel is a biocompatible substance with antibacterial capabilities, diminishing the necessity for anaesthetic during medical procedures. Moreover, it selectively targets and eliminates solely the damaged tissue while concurrently enhancing the preservation of surrounding healthy tissue [38]. The suggested mechanism postulates that the proteolytic activity of papain gel initiates chemical debridement, resulting in the breakdown and removal of the fibrin mantle generated during the carious process. The process of collagen molecule degradation occurs after perished cells are digested [39]. The collagen that has been impaired is subjected to chlorination by chloramines, resulting in the release of oxygen and subsequent bubbling and bleaching of the gel. The dentin that has undergone chemical softening is removed using a pendulum instrument until the cavity appears transparent. This process disrupts the hydrogen bonding and impacts the secondary and quaternary structure [17, 28].

Caries-detecting dyes have been employed in caries eradication to distinguish between clinically “infected” and “affected” dentin. Caries-indicator dyes have been proposed as a potential substitute diagnostic tool for occlusal caries. The protein dyes mentioned in this study can stain the organic matrix of dentin with lower mineralization levels. This includes both normal circumpulpal dentin and sound dentin located in the region of the amelo-dentinal junction [40].

Nevertheless, numerous investigations have indicated that the dye fails to effectively differentiate between bacterially infected tissues and those affected by softening. According to Banerjee et al. [16], dyes are not commonly recommended for lesions penetrating the middle third of dentin or beyond. This is because there is a higher chance of causing pulpal involvement during cavity preparation, which could be both unneeded and preventable.

Manufacturers advise repeating the treatment process until there is no occurrence of dentine discolouration. Nevertheless, the application of these fluid dyes has the potential to disperse and affect neighbouring or adjacent restorations. The term “sable” refers to a small carnivorous mammal belonging to the Must Stains, which can cause both carious and non-mineralized dentin to exhibit a dark green colouration. The pigments have a preferential affinity for substances that possess a significant amount of organic matter. As a result, this substance’s imprudent utilization might result in excessive tissue preparation, which may promote the unneeded removal of dentin over the pulpal surface and lead to an excessive removal at the enamel-dentine junction [41].

Using bulk-fill composites offers several benefits, including decreased time requirements and prevention of air spaces that can negatively impact the material properties when using traditional incremental application methods. Bulk-fill composites exhibit enhanced translucency and employ distinct photo-initiator methods to ensure sufficient conversion rates when applied in greater thicknesses than traditional incremental composites. In addition, it is worth noting that the resin matrix found in numerous bulk-fill composites incorporates resins specifically designed to absorb contraction stress, mitigating interfacial contraction stresses [42].

According to FDI criteria (aesthetic, functional, and biological) in terms of colour stability and transparency, better colour match, as well as no discrepancy in transparency and shade, was achieved for 100% of cases under BRIX3000 & rotary method at 1, 3, and 6 months when compared to caries detecting dyes in the current study. Harorli et al. show that inattention to CDD use might result in visible colour changes in tooth-coloured restorative materials [43]. Physiological wear equal to enamel was reported in all groups. The benefit of bulk-fill resin composites is their low modulus of elasticity, which decreases polymerization shrinkage stress within the composite material. However, more significant deformation and fatigue wear on the restoration and the residual tooth structure under load would occur [40]. There was no significant difference in hypersensitivity and vitality among all BRIX3000 patients compared to caries-detecting dyes and the rotary group. No secondary or primary caries, full integrity, or healthy mucosa close to repair were seen regardless of approach. Under both occlusal and proximal caries, BRIX3000 is showing better results numerically for most of the aesthetic, functional, and biological properties, but the difference was not statistically significant. The future study needs to be done with a larger sample. Moreover, discussing the results and their repercussions from the broadest conceivable perspective is essential. It may also emphasize potential future research directions.

## Conclusions

In general, the rotary method exhibited the highest mean volume of the cavity, while the BRIX 3000 group had the lowest mean volume compared to caries-detecting dyes. Within this study’s constraints, CMCR (BRIX3000) is more effective at preserving dentin, which will increase the chance of preserving the pulp’s vitality.

## Data Availability

The datasets used and analyzed during the current study are available from the corresponding author upon reasonable request.
